# Optimization of Human Neural Progenitor Cells for an Imaging-Based High-Throughput Phenotypic Profiling Assay for Developmental Neurotoxicity Screening

**DOI:** 10.3389/ftox.2021.803987

**Published:** 2022-02-16

**Authors:** Megan Culbreth, Johanna Nyffeler, Clinton Willis, Joshua A. Harrill

**Affiliations:** ^1^ Center for Computational Toxicology and Exposure, Office of Research and Development, U.S. Environmental Protection Agency, Durham, NC, United States; ^2^ Oak Ridge Institute for Science and Education (ORISE) Postdoctoral Fellow, Oak Ridge, TN, United States

**Keywords:** developmental neurotoxicology, high-throughput (HT) approaches, phenotypic profiling, human-derived cells, computational toxicology

## Abstract

Studies in *in vivo* rodent models have been the accepted approach by regulatory agencies to evaluate potential developmental neurotoxicity (DNT) of chemicals for decades. These studies, however, are inefficient and cannot meet the demand for the thousands of chemicals that need to be assessed for DNT hazard. As such, several *in vitro* new approach methods (NAMs) have been developed to circumvent limitations of these traditional studies. The DNT NAMs, some of which utilize human-derived cell models, are intended to be employed in a testing battery approach, each focused on a specific neurodevelopmental process. The need for multiple assays, however, to evaluate each process can prolong testing and prioritization of chemicals for more in depth assessments. Therefore, a multi-endpoint higher-throughput approach to assess DNT hazard potential would be of value. Accordingly, we have adapted a high-throughput phenotypic profiling (HTPP) approach for use with human-derived neural progenitor (hNP1) cells. HTPP is a fluorescence-based assay that quantitatively measures alterations in cellular morphology. This approach, however, required optimization of several laboratory procedures prior to chemical screening. First, we had to determine an appropriate cell plating density in 384-well plates. We then had to identify the minimum laminin concentration required for optimal cell growth and attachment. And finally, we had to evaluate whether addition of antibiotics to the culture medium would alter cellular morphology. We selected 6,000 cells/well as an appropriate plating density, 20 µg/ml laminin for optimal cell growth and attachment, and antibiotic addition in the culture medium. After optimizing hNP1 cell culture conditions for HTPP, it was then necessary to select appropriate in-plate assay controls from a reference chemical set. These reference chemicals were previously demonstrated to elicit unique phenotypic profiles in various other cell types. Aphidicolin, bafilomycin A1, berberine chloride, and cucurbitacin I induced robust phenotypic profiles as compared to dimethyl sulfoxide vehicle control in the hNP1 cells, and thus can be employed as in-plate assay controls for subsequent chemical screens. We have optimized HTPP for hNP1 cells, and consequently this approach can now be assessed as a potential NAM for DNT hazard evaluation and results compared to previously developed DNT assays.

## Introduction

In recent years, there has been an increase in neurodevelopmental disorders (Zablotsky et al., 2019). The precise etiology of this increase is unclear, but developmental exposure to man-made chemicals is suspected to contribute (Grandjean and Landrigan 2014). As such, there is a need to efficiently and reliably evaluate chemicals for developmental neurotoxicity (DNT) hazard. DNT is defined as any adverse effect resulting from chemical exposure(s) on the normal development of the nervous system (NAFTA-TWG 2016). Regulatory agencies have historically relied on *in vivo* rodent models to ascertain the DNT hazard potential of chemicals (USEPA 1998; OECD 2007). These guideline studies, however, are time-intensive, expensive, and require a large number of animals for chemical safety testing which has ethical considerations. Moreover, translation of rodent data to humans can have many uncertainties and may not be straightforward (Tsuji and Crofton 2012).

In order to overcome the limitations of these traditional *in vivo* studies, a battery of new approach methods (NAMs) which rely on *in vitro* models has been proposed. This battery includes assays that examine fundamental cellular processes (e.g., proliferation, differentiation, apoptosis) as well as those unique to neurodevelopment (e.g., neurite outgrowth, synaptogenesis, network formation) (Sachana et al., 2019). Furthermore, several of these DNT NAMs incorporate human-derived cell models (Balmer et al., 2012; Krug et al., 2013; Baumann et al., 2016; Druwe et al., 2016; Nyffeler et al., 2017; Harrill et al., 2018). Although the battery intends to maximize efficiency of DNT hazard evaluation, the need for multiple assays to assess each developmental process can delay the prioritization of chemicals for further in-depth analyses. Moreover, these assays are presently performed in low- to medium-throughput formats, which limit the chemical screening capacity. Therefore, a more broad-based higher-throughput approach to evaluate the DNT hazard potential of chemicals is merited.

The recent Next Generation Blueprint for Computational Toxicology at the United States Environmental Protection Agency (U.S. EPA) promotes the use of broad-based approaches for hazard evaluation, particularly as an initial step to characterize biological activity of chemicals and define potency thresholds for biological effect(s) (Thomas et al., 2019). High-throughput phenotypic profiling (HTPP) is one such approach. Our laboratory has previously adapted a Cell Painting (CP) assay (Bray et al., 2016) for HTPP in human-derived cell models (Nyffeler et al., 2020; Willis et al., 2020). This fluorescence-based imaging assay allows for simultaneous visualization of multiple cellular organelles (i.e., nucleus, nucleolus, endoplasmic reticulum, actin cytoskeleton, Golgi apparatus, plasma membrane, mitochondria) and extraction of individual cell-level features (e.g., position, morphology, intensity, texture). Thus far, this approach has not been established for any neuronal cell type. We therefore wanted to optimize HTPP in a human neural progenitor cell model, so that we may evaluate the utility of this approach for DNT hazard assessment. To do this, however, presented several key challenges which included 1) selecting an appropriate cell model, 2) optimizing cell culture conditions (e.g., plating density, growth surface substrates, media constituents), and 3) identifying suitable in-plate assay control chemicals.

We selected the hNP1 human neural progenitor cells as the initial model for optimizing HTPP experimental conditions in a neuronal cell type. The hNP1 cells are derived from a neuroepithelial lineage of human embryonic stem cells and express the intermediate filament protein nestin (Dodla et al., 2011). These cells have previously been utilized in two U.S. EPA DNT battery assays, proliferation and apoptosis, and are amenable to high-content image analysis (Harrill et al., 2018). This prior work with the hNP1 cells was in 96-well plate format, involved manual processing of samples, and thus was medium-throughput. To enhance efficiency and reduce cost, our HTPP approach utilizes a 384-well plate format and several automated processes (Nyffeler et al., 2020). Therefore, we had to determine an appropriate plating density and optimize the high-throughput automation for the hNP1 cells. For HTPP, we define an appropriate plating density as one where 1) cells do not become overly confluent within the assay timeframe, allowing for accurate cell segmentation, and 2) cells are not too sparse, allowing for optimal cell growth and measurement of a reasonable number of cells per well which does not overwhelm available data analysis and storage capabilities. This was also the first cell line our laboratory has implemented for HTPP that requires additional growth surface substrates. Thus, we had to establish an automated 384-well plate coating procedure.

In addition, the hNP1 cells are typically grown on 20 µg/ml laminin coated surfaces (Harrill 2018). This is a relatively high concentration and would be expensive to implement for HTPP. Therefore, we performed a laminin titer to ascertain the minimum concentration required for optimal cell growth and attachment. The hNP1 cells are also normally cultured in antibiotic-free conditions (Druwe et al., 2015; Harrill et al., 2018). This can be a liability for HTPP as inspection of thousands of individual wells for cell culture contamination is not feasible. Thus, we wanted to determine if antibiotic addition to the culture medium had any effect on the cellular phenotype. For the abovementioned experiments, culture conditions that yielded roughly the same phenotypic profile as relevant controls were considered optimal for the hNP1 cells.

Finally, we evaluated a set of candidate phenotypic reference chemicals that included 20 test chemicals, two negative controls, and one cell viability control. These chemicals were not necessarily specific for the hNP1 cells, but have been demonstrated to induce diverse phenotypic profiles in various cell types (Gustafsdottir et al., 2013; Nyffeler et al., 2020; Willis et al., 2020). Our aim was to identify appropriate in-plate assay controls for future HTPP DNT chemical screens in the hNP1 cells. Each chemical was tested over a concentration range in both cell viability (CV) and CP assays to obtain respective phenotypic profiles. Those chemicals that induced a marked effect on cellular phenotype relative to dimethyl sulfoxide (DMSO) vehicle control at non-cytotoxic concentrations were considered appropriate as in-plate assay controls for the hNP1 cells.

## Materials and Methods

### Cell Culture

Laboratory stocks of the hNP1 cells, originally purchased from ArunA Biomedical, were used for the present experiments. Passage 6 (P6) cells were expanded to P8 on poly-l-ornithine (PLO) (final concentration 10 µg/ml; Sigma) and laminin (final concentration 20 µg/ml; Sigma) pre-coated T75 flasks (Corning) in hNP1 proliferation medium [Knockout™ DMEM/F-12 supplemented with StemPro^®^ Neural Supplement (1X), GlutaMAX™ (1X), and human EGF and FGF-basic recombinant proteins (final concentration 20 ng/ml); GIBCO]. Cells were then cryopreserved in hNP1 proliferation medium plus 10% DMSO (Sigma) at 3 × 10^6^ cells/mL, and stored in vapor phase liquid nitrogen. For all experiments, the P8 cells were rapidly thawed at 37°C and cultured as described above. Flasks were incubated in a 37°C, 5% CO_2_ humidified incubator and media was changed after 24 h and every 2–3 days thereafter. Cells reached 80–90% confluency approximately 8 days from thaw and 5 days from subculture when seeded at 4 × 10^4^ cells/cm^2^, then were detached with TrypLE™ Select Enzyme (GIBCO) and centrifuged at 264 × g for 5 min to obtain a cell pellet. The pellet was resuspended in hNP1 proliferation medium and a subsample removed for analysis of trypan blue (0.4%; Invitrogen) exclusion with a Neubauer chamber to assess cell viability and relative cell count. On average, the hNP1 cells were 80% viable from thaw. At P10, cells were plated (6,000 cells/well unless otherwise specified) on pre-coated CellCarrier-384 Ultra Microplates (384CC; Perkin Elmer, Waltham, MA, United States) in 40 µL hNP1 proliferation medium with or without Penicillin-Streptomycin (1%; HyClone 10,000 U/mL Penicillin, 10,000 µg/ml Streptomycin) using a CERTUS FLEX Micro Dispenser (CERTUS; Trajan Scientific, Carrboro, NC, United States) equipped with a 0.45/0.15 mm microvalve. See below for details on the 384CC plate pre-coat procedure.

Tissue culture-treated 384CC plates were labelled with unique barcodes prior to the pre-coat procedure. PLO (final concentration 10 µg/ml) diluted in double distilled water (ddH_2_O) was added at 40 µL/well using the CERTUS equipped with a 0.30/0.10 mm microvalve. The plates were then wrapped with plastic wrap and stored at 4°C typically for 72 h. Note that a minimum of 24 h and a maximum of 96 h achieved similar results; however, shorter or longer storage conditions were not examined. Prior to cell plating, PLO was removed, and the plates were washed one time with ddH_2_O using a CyBio FeliX (Felix; Analytik Jena) automated pipette robot. Finally, laminin (final concentration 20 µg/ml unless otherwise specified) was diluted directly in the cell culture suspension, and cells plated as described above.

### Chemical Exposure

To identify appropriate in-plate assay-specific controls, chemicals were selected that induce unique phenotypic profiles in the CP assay, but were not necessarily dependent on the cell type (Table 1). Chemicals were solubilized in DMSO (0.1–60 mM range) to create stock solutions and stored at −20°C. To prepare dose plates, stocks were thawed and select chemicals further diluted in DMSO (0.002–20 mM range) to generate appropriate top concentrations. All chemicals were then pipetted into an Echo^®^ qualified 384-well polypropylene plate (384PP; LabCyte), and the Echo^®^ 550 (Echo; LabCyte) acoustic dispenser utilized to create a dilution series for each chemical in an Echo^®^ qualified 384-well low dead volume plate (384LDV). The stocks from the 384PP plate were dispensed at varying volumes to the 384LDV plate, and this plate backfilled with varying volumes of DMSO using the CERTUS equipped with a 0.10/0.03 mm microvalve. The 384LDV plate was then sealed and stored at −80°C until use. All concentrations on the 384LDV plate were 200X the final concentration tested.

**TABLE 1:** Reference chemical set utilized to identify appropriate in-plate assay controls.

Group	Chemical name	DTXSID	Putative molecular Target/Mechanism(s) of action	References	Vendor	Catalog Number	Stock Conc. (mM)	Tested concentration range (µM)	
								Minimum	maximum
Test Chemical	5,8,11-Eicosatriynoic acid	DTXSID10159018	Non-selective lipoxygenase inhibitor	Hammarström (1977)	Caymen	90200	50	0.0833	250
	Actinomycin D	DTXSID9020031	Transcription inhibitor	Sobell (1985)	Sigma	A9415	1	3.33E-04	1
	Amiodarone hydrochloride	DTXSID7037185	Non-selective ion channel blocker	Kodama et al. (1997)	Sigma	A8423	20	0.0333	100
	Amperozide	DTXSID6048416	5-HT2Areceptor antagonist	Haskins et al. (1987)	Santa Cruz	sc-203512	20	0.0333	100
	Aphidicolin	DTXSID5036711	DNA polymerase inhibitor	Ikegami et al. (1978)	Sigma	A0781	2	0.00333	10
	Bafilomycin A1	DTXSID201015547	Vacuolar ATPase inhibitor	Bowman et al. (1988)	Caymen	11038	0.1	1.67E-04	0.5
	Berberine chloride	DTXSID8024602	Mitochondrial toxicant	Pereira et al. (2007)	Sigma	B3251	20	0.0333	100
	Ca-074-Me	DTXSID50881386	Cathepsin B and L inhibitor	Montaser et al. (2002)	Sigma	C5857	20	0.001	3
	Cladribine	DTXSID8022828	Induces apoptosis via dsDNA breaks	Hirota et al. (1989)	Sigma	220467	20	0.0333	100
	Cucurbitacin I	DTXSID501015546	STAT3/JAK inhibitor	Blaskovich et al. (2003)	Caymen	14747	2	3.33E-04	1
	Cycloheximide	DTXSID6024882	Protein synthesis inhibitor	Obrig et al. (1971)	Sigma	239764	20	0.0333	100
	Cytarabine	DTXSID3022877	DNA replication inhibitor	Major et al. (1982)	Sigma	251010	20	0.00333	10
	Docetaxel	DTXSID0040464	Microtubule depolymerization inhibitor	Guéritte-Voegelein et al. (1991)	Caymen	11637	20	3.33E-05	0.1
	Ethoxyquin	DTXSID9020582	Lipid peroxidation inhibitor	Pryor et al. (1988)	Sigma	31519	20	0.0333	100
	Etoposide	DTXSID5023035	DNA Topoisomerase II inhibitor	Chen et al. (1984)	Sigma	E1383	20	0.00333	10
	Exo-1	DTXSID90893483	Exocytosis inhibitor	Feng et al. (2003)	Sigma	E8280	60	0.1	300
	FCCP	DTXSID40190494	Oxidative phosphorylation uncoupler	Benz and McLaughlin (1983)	Sigma	C2920	40	0.0333	100
	Fluazinam	DTXSID7032551	Oxidative phosphorylation uncoupler	Guo et al. (1991)	Sigma	34095	20	0.0333	100
	Lys05	DTXSID901015548	Lysosomal autophagy inhibitor	Amaravadi and Winkler (2012)	Sigma	SML2097	20	0.0333	100
	Rapamycin	DTXSID5023582	mTOR complex I inhibitor	Sabatini et al. (1994)	Sigma	R0395	2	0.00333	10
Negative Control	Saccharin	DTXSID5021251	n/a	n/a	Sigma	240931	20	0.0333	100
	Sorbitol	DTXSID5023588	n/a	n/a	Sigma	S1876	20	0.0333	100
Cell Viability Control	Staurosporine	DTXSID6041131	Non-specific protein kinase inhibitor	Rüegg and Burgess (1989)	Sigma	S5921	2	1.00E-04	10

n/a: not applicable; 5-HT2A, serotonin 2A receptor; ds, double stranded; STAT3, signal transducer and activator of transcription 3; JAK, janus tyrosine kinase.

Following cell plating, the hNP1 cells were allowed to attach and grow for 24 h at which point 200 nL of the 200X stocks on the 384LDV plate were transferred to the 384CC plates using the Echo. Well coordinates on each plate were uniquely randomized with respect to treatment (chemical and concentration). The final DMSO concentration in each well was 0.5%. Chemicals were tested at eight different concentrations (half-log spacing) with two technical replicates per plate. The concentration range tested for each chemical is listed in Table 1 and was based on previous observations from our laboratory in other non-neuronal cell types (Nyffeler et al., 2020; Willis et al., 2020; unpublished data). Saccharin and sorbitol were included as negative controls, and staurosporine as a CV positive control. Staurosporine was tested at six different concentrations (log spacing) with one technical replicate per plate; however, three technical replicates of 1 µM staurosporine were tested per plate as described (Nyffeler et al., 2020). Each plate also had 24 DMSO (0.5%) vehicle control wells.

For the plating density, laminin titer, and ± antibiotics experiments, cells were not subject to chemical exposure and grew for 48 h undisturbed. The chemical treated plates, however, were allowed to attach and grow for 24 h prior to exposure, and then grew for an additional 24 h before CV or CP assay processing.

### Live Labelling and Immunocytochemistry

The fluorescent labels utilized in the present experiments were previously described (Nyffeler et al., 2020). For the CV assay, cells were live-labelled with Hoechst 33342 (Hoechst; Invitrogen) and propidium iodide (PI; Invitrogen) 24 h after chemical exposure. Hoechst and PI diluted in hNP1 proliferation medium were added at 2 µL/well using the CERTUS equipped with a 0.10/0.03 mm microvalve. Final in-plate concentrations were 7.7 and 3.6 µM, respectively. Plates were then incubated in a 37°C, 5% CO_2_ humidified incubator for 30 min. After live-labelling, cells were fixed by adding 12 µL 16% paraformaldehyde (PFA) (final concentration 3.6%; Electron Microscopy Sciences) to each well with the MultiFlo FX Microplate Dispenser (MultiFlo; BioTek) and incubated in the dark at room temperature for 10 min. Plates were then washed two times with 1X phosphate buffered saline (PBS; 10X stock from Invitrogen) using the MultiFlo, sealed with optical adhesive tape, and stored at 4°C until image acquisition.

For the CP assay, labelling was as previously described (Nyffeler et al., 2020). Briefly, cells were live-labelled with MitoTracker Deep Red (MitoTracker; Invitrogen) approximately 48 h after cell plating for the plating density, laminin titer, and ± antibiotics experiments or 24 h after chemical exposure. MitoTracker diluted in hNP1 proliferation medium was added at 2 µL/well using the CERTUS equipped with a 0.10/0.03 mm microvalve with a final in-plate concentration of 475 nM. Plates were then incubated, fixed, and washed as described above. Cells were permeabilized by adding 10 µL 0.5% Triton X-100 (final concentration 0.1%; Sigma) with the CERTUS equipped with a 0.15/0.03 mm microvalve and incubated in the dark at room temperature for 30 min. Plates were then washed two times with 1X PBS and wells drained to a residual volume of 40 µL using the MultiFlo. The staining solution which contained Hoechst, SYTO™ 14, Concanavalin A-Alexa Fluor™ 488, Alexa Fluor™ 568 Phalloidin, and Wheat Germ Agglutinin-Alexa Fluor™ 555 (Invitrogen) in 1% bovine serum albumin (BSA; Sigma) diluted in 1X PBS was added at 2 µL/well using the CERTUS equipped with a 0.10/0.03 mm microvalve and plates incubated in the dark at room temperature for 30 min. Final in-plate concentrations for each of these labels were 1.93 µM, 0.86 µM, 28.6 µg/ml, 11.8 nM, and 1.43 µg/ml, respectively. Plates were then washed four times with 1X PBS using the MultiFlo, sealed with optical adhesive tape, and stored at 4°C until image acquisition.

### Imaging and Feature Extraction

Prior to image acquisition, plates were allowed to equilibrate to room temperature. Fluorescent images were acquired using the Opera Phenix™ High-Content Screening System (Phenix; Perkin Elmer) and Harmony^®^ v4.9 (Perkin Elmer) software. For the CV assay, images were captured using a 10X air objective with four unique fields-of-view. The z-offsets for Hoechst and PI were optimized by examining DMSO and 0.1 µM staurosporine wells across multiple plates (this being the highest concentration of staurosporine tested in which not all cells were dead), and remained constant throughout all experiments. Exposure times and laser power settings for each fluorophore were optimized using DMSO vehicle control wells. Image processing with the Harmony^®^ software was as previously described (Nyffeler et al., 2020). Briefly, nuclei were segmented in the Hoechst channel, valid nuclei selected based on size and Hoechst intensity, and PI intensity measured for each valid nucleus. The cell-level data for each plate were then exported for further analysis.

For the CP assay, images were captured using a 20X water immersion objective with five unique fields-of-view. The z-offsets for each fluorophore were optimized by examining untreated wells across multiple plates, and remained constant throughout all experiments. Exposure times and laser power settings for each fluorophore were optimized using untreated or DMSO vehicle control wells. Image processing with the Harmony^®^ software was as previously described (Nyffeler et al., 2020) with minimal modifications. The positional information from nuclei segmented in the Hoechst channel was used to segment cells in the Alexa Fluor™ 568 Phalloidin channel. Cells with low intensity in the Hoechst channel or that touched the image border were excluded. Modules within the Harmony^®^ software were then used to quantify a variety of cellular features, which included but were not limited to intensity, texture, shape, and position. A total of 1,300 features were extracted for each cell. Approximately 800 cells total in five fields-of-view were analyzed for hNP1 cells seeded at 6,000 cells/well. The well-level and cell-level data for each plate were exported for further analysis.

### Data Analysis and Statistics

The number of analyzed cells/well and percent confluence were determined from well-level data. Intact Hoechst-positive cells within the five fields-of view were quantified as the number of analyzed cells/well, while percent confluence was calculated as the total area of the cell bodies in the Alexa Fluor™ 568 Phalloidin channel divided by the total area imaged multiplied by 100. Data were combined for all plates, but no within-plate or across-plate normalization was performed. The *tidyverse* (v.1.3.0) package (Wickham 2016; Wickham et al., 2019) in R statistical software (v.3.6.3) (R_Core_Team 2020) was used to graph these endpoints. Boxplots represent the median and interquartile range of the data. To ascertain statistical difference, data normality (Shapiro-Wilk) and equality of variances (Levene’s Test) (Fox and Weisberg 2019) were first determined. All data were either not normally distributed or of unequal variance; therefore, the non-parametric Kruskal-Wallis one-way analysis of variance with a posthoc Dunn’s Test with Bonferroni correction (Dinno 2017; Ogle et al., 2021) was applied. Statistical significance was set at an adjusted *p*-value < 0.05.

For the CV and CP assays, cell-level data were normalized and aggregated to the well-level using R statistical software as previously described (Nyffeler et al., 2020). Two well-level endpoints, normalized cell count and percent PI-positive cells, were calculated from cell-level CV data. The normalized cell count was the number of valid cell nuclei divided by the median number of valid cell nuclei of DMSO vehicle controls multiplied by 100; percent PI-positive cells was the number of PI-positive cells per well divided by the number of valid cell nuclei multiplied by 100. DMSO vehicle control wells with a normalized cell count less than 50% and chemical exposed wells that had less than 50 valid nuclei were excluded. Cell-level CP data were normalized to DMSO vehicle control using the normalized median absolute deviation (nMAD) (Bray et al., 2016). The median of normalized cell-level data was then quantified and scaled to the nMAD of DMSO vehicle control wells. Thus, well-level CP data represent the number of standard deviations each feature was above or below the median of DMSO vehicle control. For principal component analysis (PCA), heatmap generation, and individual positional feature (average distance and percent contact area) graphs, well-level data were used. The PCA and individual positional features were plotted with the *tidyverse* package, and statistical analysis of individual positional features was as described above. Average distance was expressed as the absolute value of the distance between the centroids of nearest neighbor cell nuclei, while percent contact area was the absolute value of the contact area of nearest neighbor cell bodies multiplied by 100.

The *tcplfit2* (v.0.1.1) (Sheffield 2021) package in R statistical software was used for concentration-response modeling of well-level CV data. Normalized cell count was fit to two functions, constant and Hill, whereas, cytotoxicity (percent PI-positive cells) was fit to three functions, constant, Hill, and Gain-Loss. The best-fit model was then selected based on the lowest Akaike information criterion (AIC) (Akaike 1974). The benchmark concentration (BMC) for each chemical and endpoint was estimated from data fit with Hill or Gain-Loss functions. The half maximal effective concentration (EC50) was calculated as the BMC for normalized cell count, while three times the nMAD was set as the benchmark response for cytotoxicity. The overall cell viability BMC was the lower concentration of the aforementioned endpoint values. As such, the lowest observable effect concentration (LOEC) was defined as the lowest concentration tested above the cell viability BMC. For subsequent well-level CP data analysis, all concentrations above the cell viability LOEC were excluded.

Feature-level and global concentration-response modeling of well-level CP data were performed as previously described (Nyffeler et al., 2021). For feature-level modeling, the *tcplfit2* package in R statistical software was used to model each of the 1,300 features to nine functions, constant, Hill, poly1, poly2, power, and exp2-5. The best-fit model was then selected based on the lowest AIC. The benchmark response for feature-level data was set as one times the nMAD. For global modeling, the Mahalanobis distance approach was applied. PCA was used to transform well-level CP data, and estimate a covariance matrix. The distance of each well from the mean position of the DMSO vehicle control wells was then calculated from the matrix. A larger Mahalanobis distance meant the profile of a well was very different from DMSO vehicle control. Again, *tcplfit2* and the abovementioned functions were used to model the global Mahalanobis distance, and the best-fit model selected based on the lowest AIC. The global or phenotypic profiling benchmark response was also set as one times the nMAD.

Raw and processed data for the present work are freely accessible *via* FigShare (DOI: 10.23645/epacomptox.16695265).

## Results

### Experimental Design for HTPP of hNP1 Cells

**FIGURE 1:**
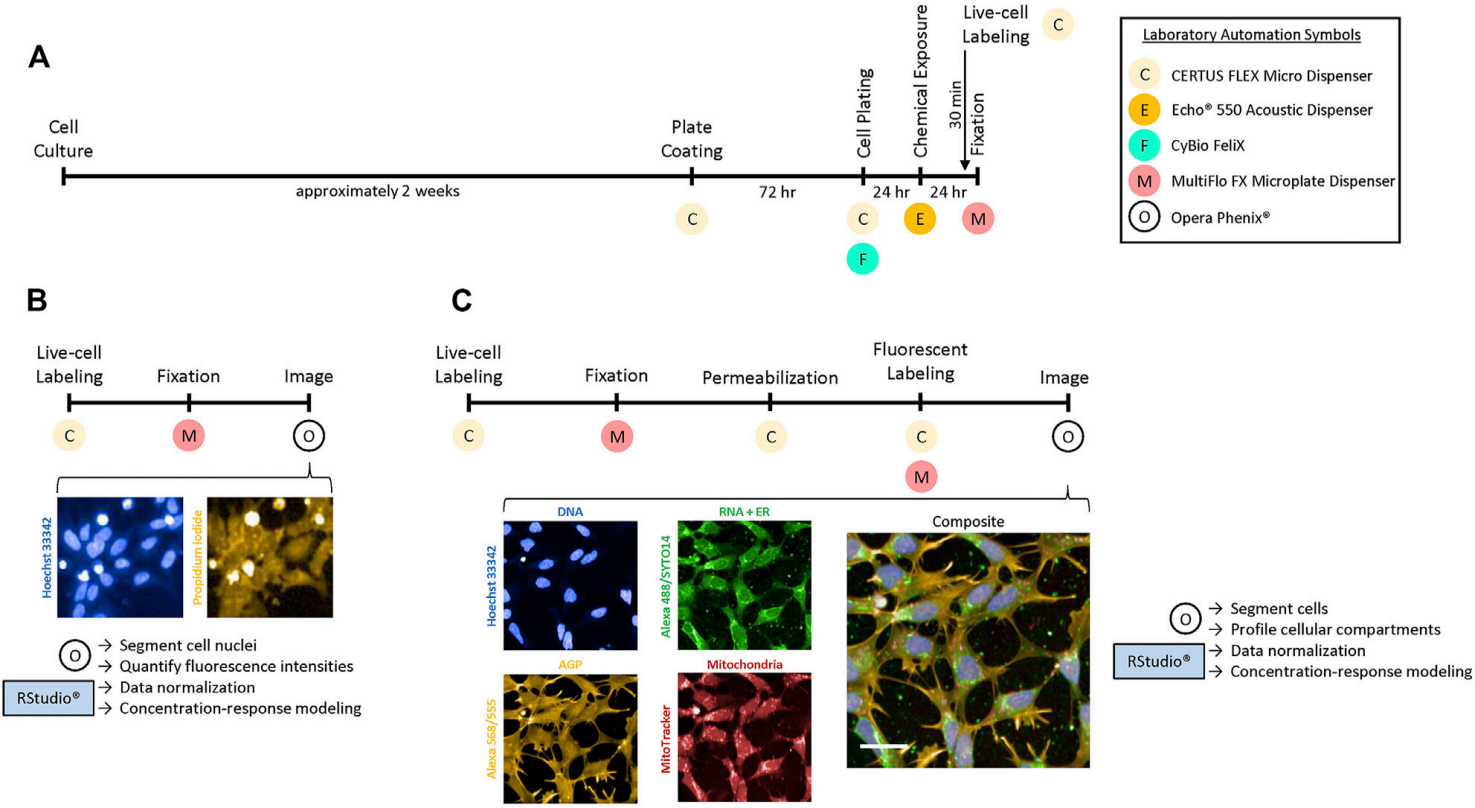
Experimental design for HTPP in hNP1 cells. **(A)** Timeline to prepare cells for the Cell Viability [CV; **(B)**] or Cell Painting [CP; **(C)**] assay. The live-cell labels were added 30 min prior to the end of the 24 h chemical exposure period. Necessary laboratory automation is denoted below each process. Images were captured with a 10X air or 20X water immersion objective for the CV or CP assay, respectively. Harmony^®^ software was used to process the images and Microsoft PowerPoint to crop and enlarge distinct areas. The cell- or well-level data obtained from the images were then exported for downstream analysis with R statistical software. Abbreviations: Concanavalin A-Alexa Fluor™ 488 (Alexa 488), Alexa Fluor™ 568 Phalloidin (Alexa 568), Wheat Germ Agglutinin-Alexa Fluor™ 555 (Alexa 555). Scale bar = 20 µm.

To process cells for HTPP requires several automation steps in order to efficiently produce reliable data. The hNP1 cells, however, have not been previously utilized in this manner. Therefore, we had to determine if these cells were compatible with the basic automation. Moreover, we also had to establish an automated 384-well plate coating procedure, as the hNP1 cells are the first cell line we have employed for HTPP that requires additional growth surface substrates. Figure 1 outlines the timeline we have instituted to process the hNP1 cells for HTPP. In the plate coating step, we elected to only pre-coat with PLO, as laminin pre-coat did not generate high quality samples for HTPP analysis in this plate format (data not shown). Cell culture, cell plating, chemical exposure, live-cell labelling, and fixation proceeded as described (Figure 1A; see *Materials and Methods*). It must be noted that in this workflow the CERTUS, Felix, Echo, and MultiFlo were not contained in sterile environments; thus, there was potential for cell culture contamination.

As mentioned, only chemical-exposed plates were processed for the CV assay (Figure 1B). Plates were typically imaged within 24 h after fixation. These representative images are of 0.01 µM staurosporine, a concentration below the BMC for cytotoxicity (Supplementary Figure S4). Hoechst-labelled cell nuclei are pictured in blue (Figure 1B; left panel), while PI-labelled cells are pictured in yellow (Figure 1B; right panel). All other experiments were processed for the CP assay (Figure 1C). Plates were typically imaged within 24 h after fluorescent labelling. These representative images are of untreated cells. Hoechst-labelled cell nuclei (Figure 1C; blue; left top panel) were utilized to profile the DNA or nuclear channel. The RNA or nucleolar and endoplasmic reticulum (ER) channels were profiled from SYTO™ 14 and Concanavalin A-Alexa Fluor™ 488 labelling, respectively (Figure 1C; green; middle top panel). The actin cytoskeleton, Golgi apparatus, and plasma membrane (AGP) channel was profiled from Alexa Fluor™ 568 Phalloidin in combination with Wheat Germ Agglutinin-Alexa Fluor™ 555 labelling (Figure 1C; yellow; left bottom panel). Mitochondria were profiled from live-labelling with MitoTracker (Figure 1C; red; middle bottom panel). A composite image with all organelle-specific fluorophores is shown (Figure 1C; right panel). Qualitatively, the hNP1 cells tended to have a larger nuclear area relative to the total cellular size. Nucleoli (RNA) were less distinct as compared to some non-neuronal cell types (Willis et al., 2020), while the ER was most prominent in the perinuclear space. The Golgi apparatus and actin cytoskeleton were also lesser defined relative to some other cell types (Willis et al., 2020), but the plasma membrane was visibly apparent. Mitochondria likewise were most intense in the perinuclear space. CV and CP data were analyzed as described (see Materials and Methods—Data Analysis and Statistics).

### Plating Density Determination

**FIGURE 2:**
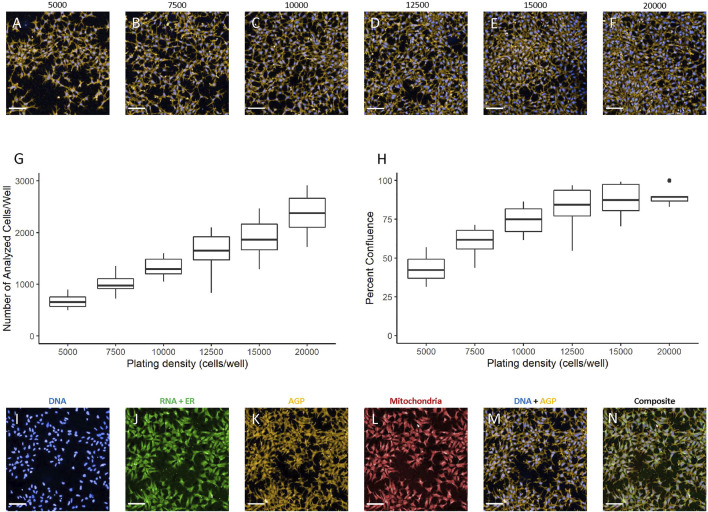
Plating density determination. Images were captured with a 20X water immersion objective and Harmony^®^ software was used for further processing. A representative image of the DNA + AGP channels at 5,000 **(A)**, 7,500 **(B)**, 10,000 **(C)**, 12,500 **(D)**, 15,000 **(E)**, and 20,000 **(F)** cells/well is displayed. Well-level data were exported to assess the number of analyzed cells/well **(G)** and percent confluence **(H)** in R statistical software. Graphs represent three biological replicates (i.e., independent cultures) which includes 16 technical replicates/density per biological replicate. Representative images at 6,000 cells/well visualized using the DNA **(I)**, RNA + ER **(J)**, AGP **(K)**, mitochondrial **(L)**, DNA + AGP **(M)**, and all channels combined **(N)** are provided. Scale bar = 100 µm.

The hNP1 cells had not previously been plated in 384-well plate format in our laboratory. Therefore, we had to determine an appropriate plating density for these cells. To select an optimal density range, we extrapolated from data for the hNP1 cells in 96-well plate format (Druwe et al., 2015). Accordingly, 5,000 cells/well was assessed as the lower limit in our plating density experiments. The hNP1 cells were plated at six different densities and plates were processed for the CP assay. Representative images of each plating density are shown (Figures 2A–F). Only Hoechst and Alexa Fluor™ 568 Phalloidin labelling are displayed, as these fluorophores alone were used to quantify the number of analyzed cells/well (Figure 2G; Supplementary Figure S1A) and percent confluence (Figure 2H; Supplementary Figure S1B). For this study, we targeted a minimum of 500 cells analyzed in five fields-of-view and 60–70% confluence. This should allow for appropriate cell segmentation and subsequent modeling of extracted cell-level features. Therefore, we interpolated 6,000 cells/well as an appropriate plating density for the hNP1 cells. This density results in approximately 800 cells analyzed in five fields-of-view and 50% confluence. As the hNP1 cells tend to grow in dense clusters, we chose a less confluent density in order to properly segment individual cells. Representative images of each channel, as well as a Hoechst-Alexa Fluor™ 568 Phalloidin composite and a composite with all organelle-specific fluorophores is shown (Figures 2I–N). For all subsequent experiments, cells were plated at 6,000 cells/well.

### Laminin Titer

**FIGURE 3:**
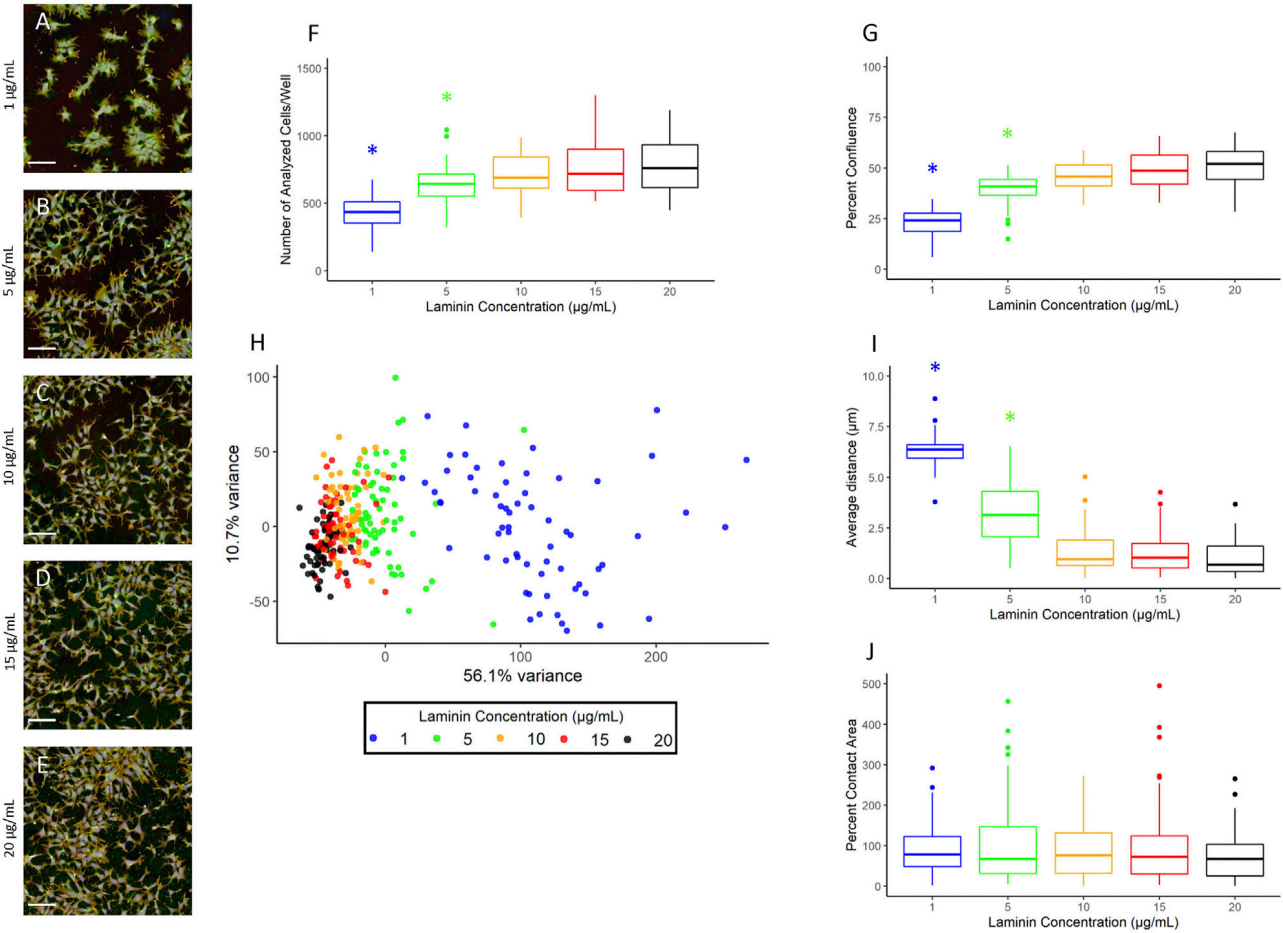
Laminin titer. Images were captured with a 20X water immersion objective and Harmony^®^ software was used for further processing. A representative composite image of the hNP1 cells cultured on 1 **(A)**, 5 **(B)**, 10 **(C)**, 15 **(D)**, and 20 **(E)** µg/mL laminin is displayed. Well-level data were exported to assess the number of analyzed cells/well **(F)** and percent confluence **(G)**, whereas cell-level data were exported for the PCA **(H)** and evaluation of individual positional features **(I,J)**. Graphs represent four biological replicates (i.e., independent cultures) which includes 16 technical replicates/concentration per biological replicate. The *x*- and *y*-axes on the principal component graph depict the variance in the first and second principal component, respectively. All statistical comparisons were made to 20 µg/ml (adjusted *p*-value, *p < 0.05). Scale bar = 100 µm.

The hNP1 cells are typically grown on 20 µg/ml laminin coated surfaces (Harrill 2018). This is a relatively high concentration and would be expensive to implement in high-throughput applications. Therefore, we wanted to determine whether lower laminin concentrations would yield a similar cell growth pattern as 20 µg/ml. The hNP1 cells were plated at five different laminin concentrations and plates were processed for the CP assay. Representative images of each laminin concentration are shown (Figures 3A–E). The number of analyzed cells/well (Figure 3F; Supplementary Figure S2A) and percent confluence (Figure 3G; Supplementary Figure S2B) were quantified, and each concentration was statistically compared to 20 µg/ml. There was an overall effect on the number of analyzed cells/well (*p* < 2.2 × 10^−16^) and percent confluence (*p* < 2.2 × 10^−16^); however, only 1 µg/ml (*p* = 2.7 × 10^−22^/*p* = 1.2 × 10^−30^) and 5 µg/ml (*p* = 3.3 × 10^−3^/*p* = 3.9 × 10^−7^) were significantly different from 20 µg/ml.

**FIGURE 4:**
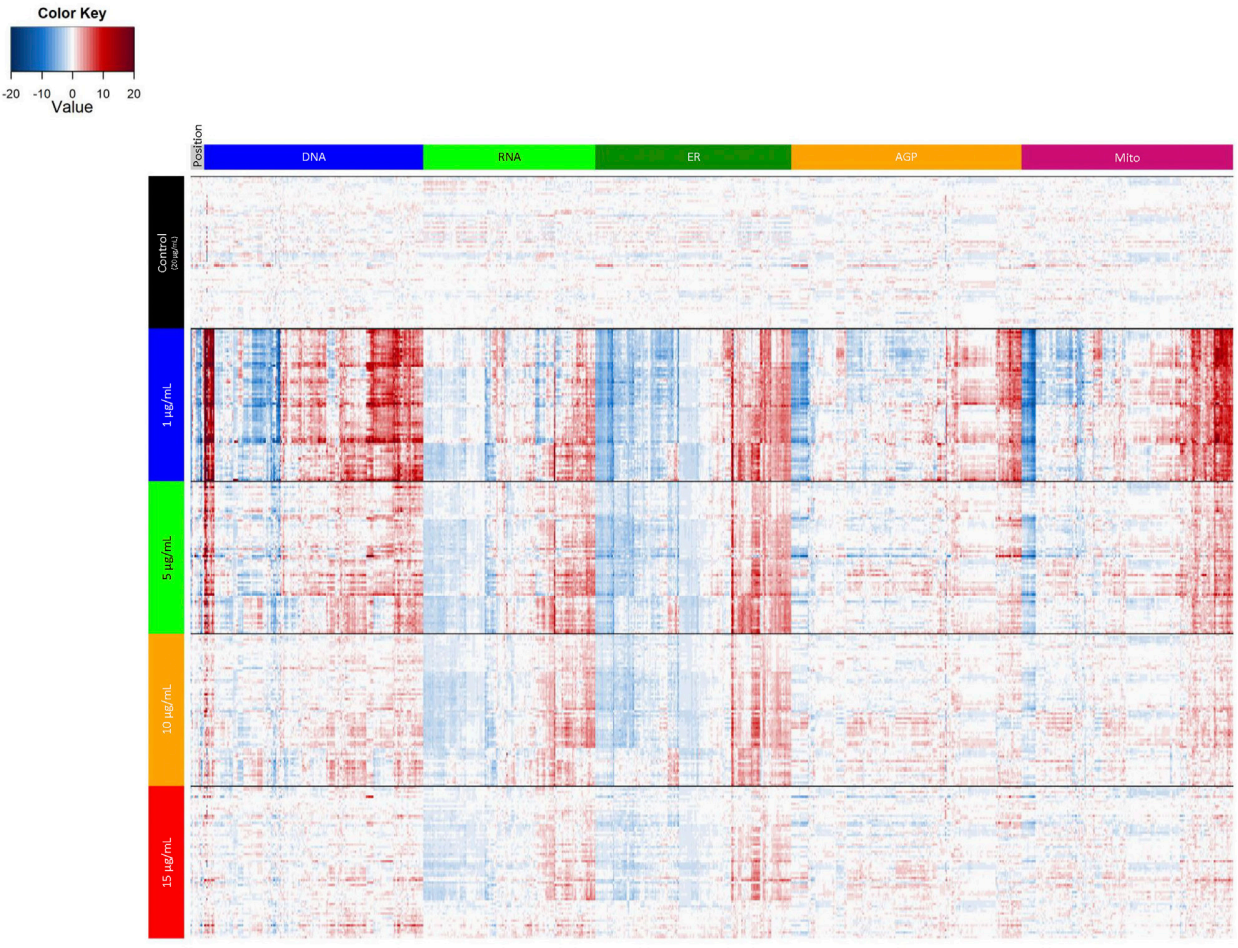
Laminin titer heatmap. Cell-level data were exported to generate the heatmap in R statistical software. Data represent four biological replicates (i.e., independent cultures) which includes 16 technical replicates/concentration per biological replicate. The rows depict individual wells and are organized by laminin concentration; the columns depict individual features and are organized by channel. Heatmap coloring represents the size and direction of the phenotypic effect relative to 20 µg/ml (Control).

To further examine potential distinctions between the different laminin concentrations, we performed a PCA on normalized well-level data (Figure 3H). These data are summarized in Figure 4. The first principal component accounted for 56.1% of the variance, and thus revealed as laminin concentration increases, the phenotypic profile clusters more tightly with 20 µg/ml. We also assessed the individual positional features “average distance” (Figure 3I; Supplementary Figure S2D) and “percent contact area” (Figure 3J; Supplementary Figure S2D). There was an overall effect on average distance (*p* < 2.2 × 10^−16^) with 1 µg/ml (*p* = 7.3 × 10^−33^) and 5 µg/ml (*p* = 9.4 × 10^−12^) significantly different from 20 µg/ml. Percent contact area was unaffected (*p* = 0.46) likely because the hNP1 cells grow in dense clusters. Importantly, clear overall phenotypic differences could be observed between 20 µg/ml and all other laminin concentrations at the level of individual feature measurements (Figure 4). Therefore, we elected to use 20 µg/ml for all subsequent experiments to ensure we were above an inflection point at 10–15 µg/ml where obvious alterations in the hNP1 cellular phenotype were apparent.

### ± Antibiotics

**FIGURE 5:**
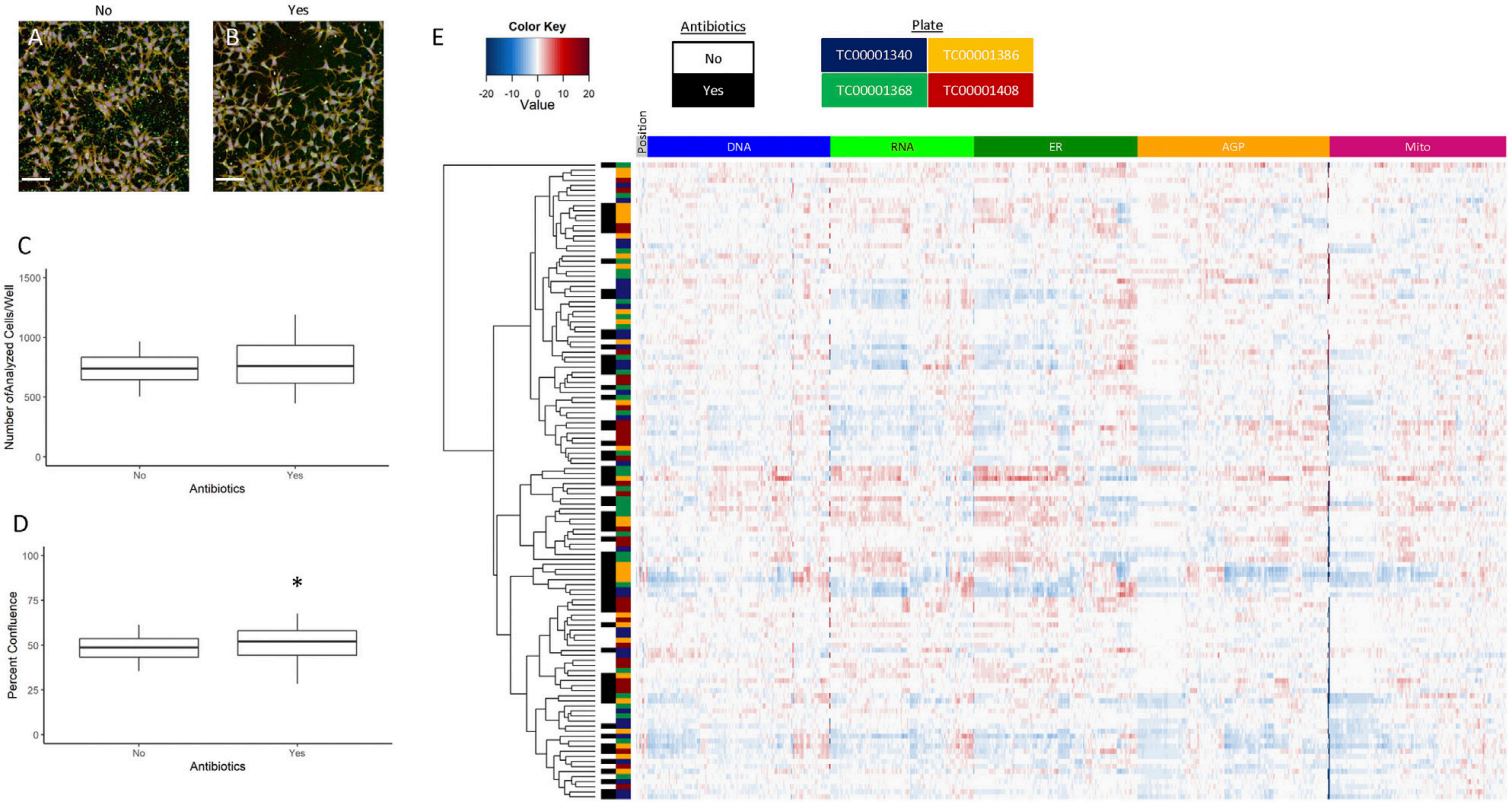
± Antibiotics. Images were captured with a 20X water immersion objective and Harmony^®^ software was used for further processing. A representative composite image without **(A)** and with **(B)** antibiotics is displayed. Well-level data were exported to assess the number of analyzed cells/well **(C)** and percent confluence **(D)**, whereas cell-level data were exported for the heatmap **(E)**. The graphs and heatmap represent four biological replicates (i.e., independent cultures) which includes 16 technical replicates/condition per biological replicate. The rows and columns on the heatmap depict individual wells and features, respectively, and is further labelled by antibiotics (no or yes) and by plate (TC0000####). Heatmap coloring represents the size and direction of the phenotypic effect relative to the no antibiotic condition. All statistical comparisons were made to “without antibiotics” (adjusted *p*-value, *p < 0.05). Scale bar = 100 µm.

The hNP1 cells are typically grown in antibiotic-free conditions (Druwe et al., 2015; Harrill et al., 2018), which is not ideal for high-throughput automated applications as inspection of thousands of individual wells for contamination is not feasible. Therefore, we wanted to determine if antibiotic addition to the culture media would alter the cell growth pattern compared to no antibiotics. The hNP1 cells were plated without (No) or with (Yes) antibiotics and plates were processed for the CP assay. A representative image without (Figure 5A) and with (Figure 5B) antibiotics is shown. The number of analyzed cells/well (Figure 5C; Supplementary Figure S3A) and percent confluence (Figure 5D; Supplementary Figure S3B) were quantified, and “with antibiotics” statistically compared to “without antibiotics.” Number of analyzed cells/well was unaffected (*p* = 0.33) by antibiotic addition. Percent confluence, however, was significantly different (*p* = 0.046).

To evaluate further potential antibiotic effects on cellular phenotype, we generated a heatmap of normalized well-level data (Figure 5E), performed unsupervised hierarchical clustering, and labeled data by antibiotics (No or Yes) and by plate (TC0000####). The data tended not to cluster by antibiotics or by plate identity. Therefore, we concluded antibiotics do not have a significant impact on the phenotypic profile of the hNP1 cells. Although, percent confluence was significantly affected, this data did not offset the lack of clustering in the profiling data. Thus, we opted to include antibiotics for all subsequent experiments. It must be noted that antibiotics were only added to the culture media for plating, and cells were grown in this condition for a maximum of 48 h. We did not examine the potential outcome of long-term growth in flasks, and do not foresee antibiotic use for general hNP1 cell culture.

### Reference Chemical Set

**FIGURE 6:**
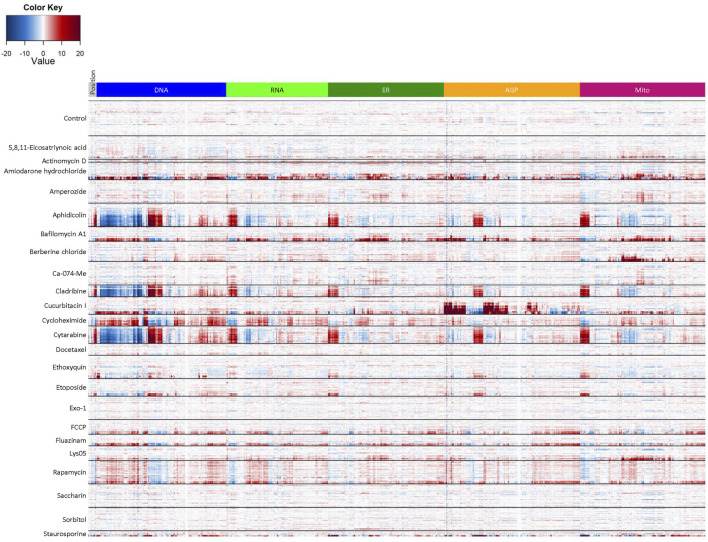
Reference chemical set heatmap. Cell-level data were exported to generate the heatmap in R statistical software. Test concentrations above the LOEC for each chemical were removed prior to analysis. Data represent three biological replicates (i.e., independent cultures) which includes two technical replicates/chemical concentration per biological replicate. The rows in each clade from top to bottom depict the tested concentration range from lowest to highest value (see Table 1) and are organized by chemical, whereas the columns depict individual features organized by channel. Heatmap coloring represents the size and direction of the phenotypic effect relative to DMSO vehicle control (Control).

Finally, we wanted to evaluate potential phenotypic reference chemicals (Table 1) to serve as in-plate assay controls for future HTPP screens in the hNP1 cells. These chemicals were not necessarily selected for any expected effects on neuronal cells or known effects on DNT relevant molecular targets, but were chosen based on the capacity to elicit unique phenotypic profiles across multiple cell types (Nyffeler et al., 2020; Willis et al., 2020; unpublished data). We first generated a heatmap of normalized well-level data (Figure 6) with tested concentrations above the LOEC for each chemical removed in order to eliminate potential confounding effects of general cytotoxicity. From these data, we were able to identify chemicals that exhibited a robust phenotypic profile as compared to DMSO vehicle control. For example, aphidicolin, cladribine, and cytarabine had qualitatively similar profiles, each yielding a clear response in the DNA channel. Moreover, berberine chloride produced effects primarily in the mitochondrial channel consistent with observations in other cell types (Gustafsdottir et al., 2013; Nyffeler et al., 2020; Willis et al., 2020). Of note, the profiles of the negative control chemicals, saccharin and sorbitol, were qualitatively similar to DMSO vehicle control.

Next, we calculated the ratio of the cell viability BMC relative to the phenotypic profiling BMC (Table 2). A larger ratio would indicate that the lowest measurable concentration which altered the phenotypic profile (i.e., phenotypic profiling BMC) was below that which altered cell viability (i.e., CV BMC), and thus the observed phenotypic effects were less likely to be concomitant with cytotoxicity. Note that the phenotypic profiling BMC was labelled as such because both CV and CP data were used to model and determine this value. Moreover, a phenotypic profiling BMC for actinomycin D could not be estimated because all tested concentrations were above the cell viability BMC.

**TABLE 2:** Benchmark concentrations for reference set chemicals.

Chemical	Benchmark concentration (µM)		
	Cell viability	Phenotypic profiling	Ratio
5,8,11-Eicosatriynoic acid	1.42E + 02	9.74E + 00	14.6
Actinomycin D	4.96E-05	—	—
Amiodarone hydrochloride	3.92E + 00	6.12E-01	6.4
Amperozide	>100	2.97E + 00	—
Aphidicolin	>10	5.35E-02	—
Bafilomycin A1	1.60E-02	2.46E-03	6.5
Berberine chloride	3.05E + 01	1.68E + 00	18.2
Ca-074-Me	>3	5.42E-02	—
Cladribine	4.65E-01	1.05E-02	44.3
Cucurbitacin I	5.76E-02	3.24E-04	177.8
Cycloheximide	9.10E-01	3.24E-02	28.1
Cytarabine	5.14E-01	1.48E-03	347.3
Docetaxel	8.94E-04	6.76E-04	1.3
Ethoxyquin	>100	1.00E + 01	—
Etoposide	5.52E-01	2.51E-01	2.2
Exo-1	>300	>300	—
FCCP	2.72E + 00	4.76E-01	5.7
Fluazinam	8.56E-01	2.95E-01	2.9
Lys05	1.60E + 00	7.89E-01	2.0
Rapamycin	5.43E + 00	1.05E-03	5,171.4
Saccharin (negative)	>100	>100	—
Sorbitol (negative)	>100	>100	—
Staurosporine (positive)	7.13E-02	3.89E-03	18.3

-: not calculable.

**FIGURE 7:**
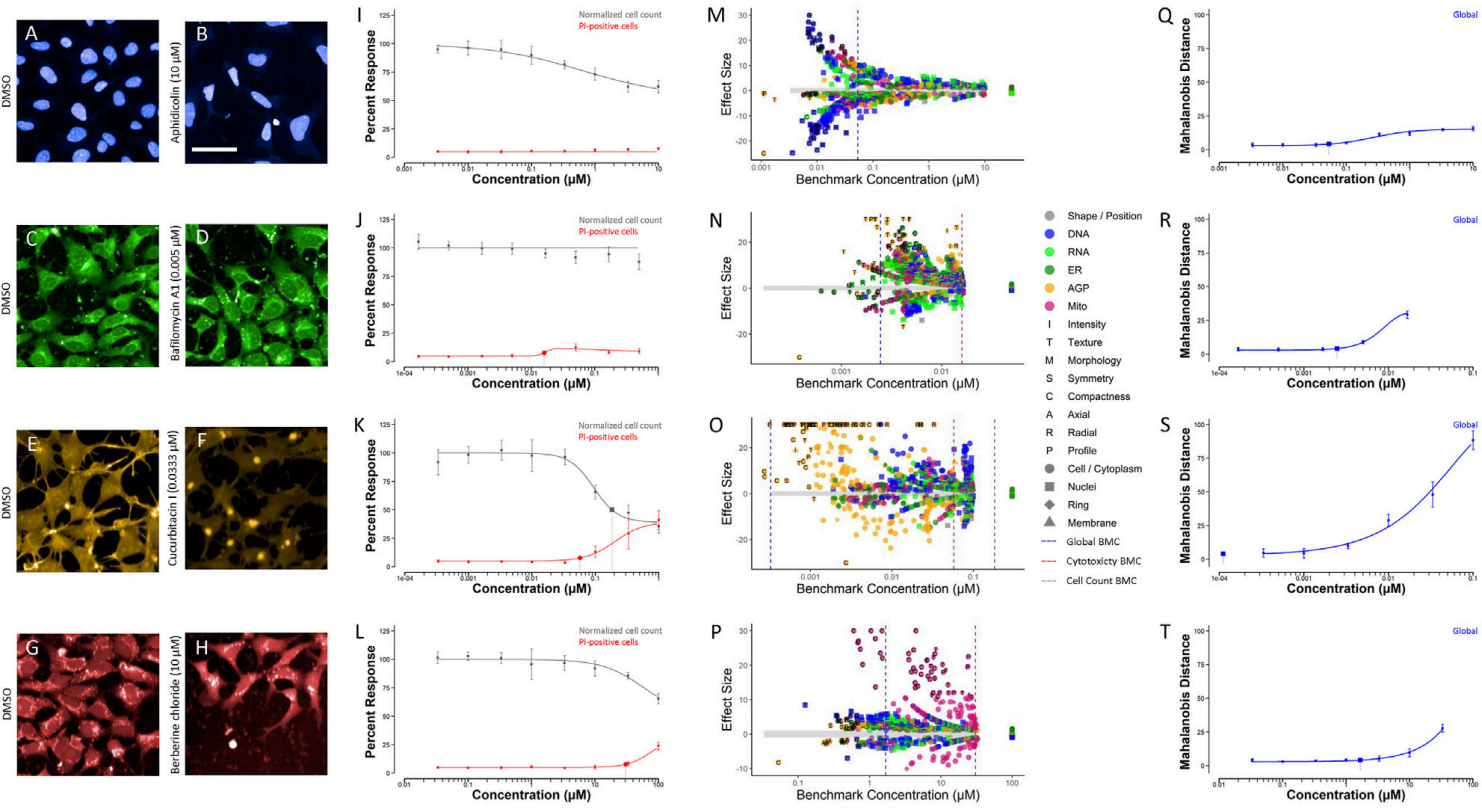
Selected reference chemicals. Images were captured with a 20X water immersion objective. Harmony^®^ software was used to process the images and Microsoft PowerPoint to crop and enlarge distinct areas. A representative image of DMSO vehicle control compared to aphidicolin **(A,B)**, bafilomycin A1 **(C,D)**, cucurbitacin I **(E,F)**, and berberine chloride **(G,H)** is displayed. Cell-level data were exported to generate cell viability **(I–L)**, feature-level potency-magnitude **(M–P)**, and global **(Q–T)** concentration-response curves in R statistical software. Data represent three biological replicates (i.e., independent cultures) which includes two technical replicates/chemical concentration per biological replicate. The enlarged symbols on the cell viability and global concentration-response curves depict the BMC for each endpoint, whereas the dotted-lines on the feature-level potency-magnitude plots depict the global (blue), cytotoxicity (red), and normalized cell count (gray) BMC, respectively. The gray band on the feature-level potency-magnitude plots represents the concentration-range modeled (i.e., all concentrations ≤ LOEC) in the horizontal direction and ±1 standard deviation from the median of the DMSO vehicle control in the vertical direction. Chemicals: aphidicolin [**(B)**: 10 μM; I: CV; **(M)**: feature-level; **(Q)**: global]; bafilomycin A1 [**(D)**: 0.005 µM; **(J)**: CV; **(N)**: feature-level; **(R)**: global]; cucurbitacin I [**(F)**: 0.0333 µM, **(K)**: CV; **(O)**: feature-level; **(S)**: global]; berberine chloride [**(H)**: 10 μM; **(L)**: CV; **(P)**: feature-level; **(T)**: global]; DMSO **(A,C,E,G)**. Scale bar = 20 µm.

An appropriate reference chemical was one that displayed a robust phenotypic profile as compared to DMSO vehicle control and had a large cell viability to phenotypic profiling BMC ratio. Based on these criteria, aphidicolin, bafilomycin A1, cucurbitacin I, and berberine chloride were identified as appropriate in-plate assay controls for HTPP conducted in the hNP1 cells (Figure 7). A representative image for each chemical alongside a DMSO vehicle control image is shown (Figures 7A–H). In addition, CV (Figures 7I–L), feature-level potencies and effect sizes (Figures 7M–P), and global concentration-response curves (Figures 7Q–T) for each chemical are displayed. Although aphidicolin had a similar phenotypic profile to cladribine and cytarabine (Figure 6), it was not cytotoxic at any concentration tested (Figure 7I). Aphidicolin induced pronounced effects in the DNA channel, particularly on nuclear compactness and radial distribution (Figures 7B,M). Unlike aphidicolin (Figure 7M), cucurbitacin I (Figure 7O), and berberine chloride (Figure 7P), bafilomycin A1 exhibited a more general effect on all channels (Figure 7N); however, it did alter cellular symmetry and texture, and radial distribution of the nuclear compartment near the phenotypic profiling BMC (dotted blue line). Cucurbitacin I produced a marked effect in the AGP channel, specifically on cellular compactness and texture (Figures 7F,O), whereas berberine chloride distinctly altered mitochondrial compactness and texture (Figures 7H,P). CV, feature-level, and global concentration-response curves for all other chemicals are provided in Supplementary Figures S4, S5, S6, respectively.

## Discussion

This study optimized experimental conditions for the broad-based, multi-endpoint HTPP approach in the hNP1 human neural progenitor cell line. It demonstrated that these cells are amenable to the laboratory automation required for HTPP, and further that plate coating with growth substrates can also be automated. Moreover, it identified an appropriate plating density for the hNP1 cells as well as the optimal laminin concentration for cell growth and attachment in 384-well plate format. This study also confirmed that antibiotics in the culture media do not alter the phenotypic profile of the hNP1 cells. Finally, aphidicolin, bafilomycin A1, cucurbitacin I, and berberine chloride were characterized as appropriate in-plate assay controls for future HTPP chemical screening as each displayed a unique and robust phenotypic profile. HTPP can now be applied for high-throughput screening of chemicals in the hNP1 cells and subsequent evaluation of assay sensitivity and predictivity of DNT hazard potential using larger chemical test sets.

The standard laminin concentration used in previous DNT assays with the hNP1 cells was 20 µg/ml. These studies were conducted in 96-well plate format in plates pre-coated with laminin (Druwe et al., 2015; Harrill et al., 2018). In a communication with an author from this prior work, it was noted that lower laminin concentrations did not yield viable hNP1 cultures (unpublished data). As an analogous coating procedure did not produce reliable growth and attachment of the hNP1 cells in 384-well plate format (data not shown), we opted to adapt an existing protocol used in microelectrode array (MEA) assay preparations that dilutes laminin directly in the cell culture suspension (Strickland et al., 2018). This protocol, however, had not previously been evaluated in the hNP1 cells. The PLO coating procedure was kept consistent with earlier DNT assays, except for the new automation component. Since 20 µg/ml laminin would be expensive to implement in HTPP, we elected to perform a laminin titer with the updated coating procedure to determine if results were comparable to previous data in 96-well plates. Although, 10 and 15 µg/ml were not significantly different for any selected endpoints in the HTPP format, the overall phenotypic profiles for these concentrations were qualitatively distinct (see Figure 4) and did not completely overlay 20 µg/ml (see Figure 3H). Moreover, these concentrations represent a potential tipping point (Radio et al., 2008) for effects on the hNP1 cellular phenotype, and thus a saturating concentration of 20 µg/ml would be more reliable for use in HTPP of these cells. Not only does this lessen potential concentration-phenotype related effects, but it also makes compiled chemical data more correlative to previous screens with the hNP1 cells. This latter type of comparison, however, is outside the scope of the present work.

The hNP1 cells were previously plated at 15,000 cells/well in 96-well plate format (Druwe et al., 2015). This approximates roughly 5,000 cells/well in 384-well plate format based on the surface area of the well. Earlier work with the hNP1 cells determined that less than 5,000 cells/well in 384-well plate format did not produce viable cultures (data not shown). Therefore, we set 5,000 cells/well as the minimum for plating density determination in 384-well plate format. As mentioned, we initially defined an optimal plating density as one that would yield at least 500 cells analyzed in five fields-of-views at 20X magnification and 60–70% confluence. These particular parameters, however, can vary between different cell types due to a diversity of sizes, shapes, and growth patterns (Willis et al., 2020). As the hNP1 cells tend to grow in dense clusters, relative confluency had a greater impact on the Harmony^®^ software capability to accurately segment individual cells (data not shown). Therefore, we optimized for a lower confluence that still achieved at least 500 cells analyzed in five fields-of-view. Technically, 5,000 cells/well reached these criteria (approximately 674 cells and 43% confluence), however, the lower range of the number of analyzed cells/well could potentially result in non-viable cultures. For this reason, we opted to marginally increase our selected plating density to 6,000 cells/well (approximately 846 cells and 56% confluence).

A recent effort to identify putative DNT negative compounds found that antibiotics may not necessarily be appropriate negative controls, as potential DNT effects *in vivo* were discerned. Moreover, effects appeared to be dependent upon the specific antibiotic under investigation (Martin et al., 2022; manuscript in preparation). Antibiotics were not previously included in the culture media for DNT assays with the hNP1 cells (Druwe et al., 2015; Harrill et al., 2018). To our knowledge, the possible impact antibiotics could have on the hNP1 cellular phenotype *in vitro* has not been systematically evaluated. Antibiotics have, however, been routinely and successfully used in previous DNT assay development with other neural progenitor cell lines (Breier et al., 2008; Culbreth et al., 2012; Druwe et al., 2015). Another consideration in establishing an HTPP assay is that antibiotic exclusion can be a liability for high-throughput applications, as inspection of thousands of individual wells for contamination is not practical. Antibiotics, however, do not entirely eliminate the risk of contamination, and thus the benefit does not necessarily outweigh potential effects on cellular phenotype. Therefore, it was imperative that we meticulously examine any possible antibiotic-related effects on the hNP1 cells. Although there was a slight, albeit significant, increase in hNP1 cell confluency, this did not offset the lack of a clear antibiotic effect on the overall phenotypic profile of these cells (see Figure 5E). Interestingly, some general phenotypic effects, which were not ascribed to antibiotics or biological variability, could be observed. We speculate these are positional effects on the plate, as wells were not randomized for these experiments. The reference chemical experiments were randomized, and future DNT screens will be as well, so we do not anticipate these positional effects had any impact on our current conclusions or will further impact our future analyses.

A chemical training set which includes known DNT compounds is often employed to identify in-plate positive controls for assay-specific neurodevelopmental endpoints (Crofton et al., 2011; Harrill et al., 2011; Druwe et al., 2015; Harrill et al., 2018). HTPP is a more broad-based, multi-endpoint approach that does not necessarily have a clear “positive” outcome, and as such, utilizes reference chemicals which reliably produce characteristic phenotypes to track assay performance. Thus, a training set limited to DNT compounds may not be optimal to identify appropriate in-plate assay controls in this case. Therefore, we compiled a reference chemical set that included compounds known to display robust and unique phenotypic profiles in a variety of cell types (Nyffeler et al., 2020; Willis et al., 2020; unpublished data). This set, however, had not previously been evaluated in any neuronal cell type. The negative controls, saccharin and sorbitol, and the cell viability positive control, staurosporine, were part of training sets for proliferation and apoptosis assays, respectively, in the hNP1 cells (Druwe et al., 2015; Harrill et al., 2018). Aphidicolin and actinomycin D were previously employed as assay positive controls in another human-derived neural progenitor cell line (Breier et al., 2008; Culbreth et al., 2012). Therefore, we selected aphidicolin as an HTPP in-plate control for the hNP1 cells relative to cladribine and cytarabine, which displayed similar phenotypic profiles (see Figure 6). Not only was aphidicolin formerly used as a DNT assay control, but it also was not cytotoxic in the concentration range tested (see Table 2). On the other hand, actinomycin D was cytotoxic at all concentrations evaluated; therefore, it was eliminated from consideration, as a non-cytotoxic phenotypic profile could not be modeled. As mentioned, the other HTPP in-plate controls, bafilomycin A1, cucurbitacin I, and berberine chloride, were selected because each displayed a robust phenotypic profile in general or in a specific channel for the hNP1 cells (see Figure 6).

Although the reference chemical set was not previously evaluated in any neuronal cell type, several of these compounds have been assessed in the HTPP assay for various other cell lines (e.g., U-2 OS, MCF7, A549) (Gustafsdottir et al., 2013; Nyffeler et al., 2020; Willis et al., 2020). As was demonstrated in the present experiments, the negative control compounds, saccharin and sorbitol, affected very few phenotypic features (see Figure 6), similar to previous results (Nyffeler et al., 2020; Willis et al., 2020). Likewise, berberine chloride reliably affected mitochondrial compactness and texture in the hNP1 cells (see Figure 7P), consistent with other cell types (Nyffeler et al., 2020; Willis et al., 2020). Rapamycin induced more general effects on all phenotypic features, however, as with prior experiments (Nyffeler et al., 2020; Willis et al., 2020), the effect size was small (see Figure 6). Thus, while rapamycin and bafilomycin A1 evoked comparable responses in the hNP1 cells, bafilomycin A1 produced a greater magnitude of effect (see Figure 6), and therefore was selected as an in-plate assay reference chemical. Interestingly, Ca-074-Me and etoposide, which were previously used as reference chemicals in the HTPP assay (Nyffeler et al., 2020), induced a vastly different phenotypic profile in the hNP1 cells; Ca-074-Me had no apparent effect on Golgi apparatus intensity and etoposide had a less robust impact on cellular size relative to prior results (see Supplementary Figure S5) (Gustafsdottir et al., 2013; Nyffeler et al., 2020; Willis et al., 2020). Neither of these chemicals have been evaluated in any other *in vitro* DNT battery assay with neural progenitor cells. Moreover, although etoposide was demonstrated to reduce neurite length, this effect was concomitant with cytotoxicity (Krug et al., 2013). Potentially, these divergent responses in the hNP1 cells are cell type specific, but this remains to be further explored.

As HTPP has been optimized for the hNP1 cells, we can now apply this approach to screen DNT relevant compounds (Mundy et al., 2015; Aschner et al., 2017; Masjosthusmann et al., 2020) in order to establish the potential utility of this assay for DNT hazard evaluation. We would then compare HTPP results to that of previous *in vitro* DNT assays, to determine whether it is a comparable or more sensitive indicator of DNT than neurodevelopmental specific endpoints, or if it improves DNT hazard prediction over, or in combination with, existing methods. It is important to note that development of the nervous system involves many different cell types (e.g., neural progenitors, glial cells, neurons of various types) and that developmental neurotoxicants may affect critical processes of nervous system development that are not recapitulated in the hNP1 cell model. Therefore, similar to other *in vitro* DNT assays that have been developed (Harrill et al., 2018; Shafer 2019; Masjosthusmann et al., 2020), it is possible that the HTPP approach in the hNP1 cells would not detect all potential developmental neurotoxicants. Comparison of screening results for DNT relevant compounds tested in the HTPP approach in the hNP1 cells to results from DNT assays addressing other aspects of nervous system development will be key to understanding how best to deploy this approach as either part of a DNT assay battery or as the first tier in a NAMs-based hazard evaluation approach (Thomas et al., 2019) focused on DNT. Either way, HTPP is a non-targeted high-throughput approach, and thus offers a potentially more efficient method to screen and prioritize chemicals for subsequent DNT testing. Since the current DNT battery necessitates multiple assays to evaluate potential toxicity (Sachana et al., 2019), a single method that detects a similar proportion of DNT chemicals would be useful in terms of testing efficiency. However, it will also be important to establish the specificity of HTPP in terms of DNT hazard prediction. In addition, we are currently optimizing the HTPP approach for a mouse neural progenitor cell line. This will not only allow for assay-level species comparisons, but also aid in determining the most appropriate model(s) for *in vitro* to *in vivo* extrapolation. In conclusion, we have established the methods necessary to apply HTPP in order to evaluate the hNP1 human neural progenitor cells, and thus developed a new approach with the potential to efficiently and reliably screen chemicals for DNT hazard.

## Data Availability

The datasets presented in this study can be found in online repositories. The names of the repository/repositories and accession number(s) can be found below: https://epa.figshare.com/articles/dataset/HTPP_Methods/16695265.

## Supplementary Material

Supplementary Figure S1 Plating density determination faceted by plate. Well-level data were exported to assess the number of analyzed cells/well **(A)** and percent confluence **(B)** in R statistical software. Graphs are faceted by plate (TC0000####) and each facet represents one biological replicate (i.e., independent culture) which includes 16 technical replicates/density.

Supplementary Figure S2 Laminin titer faceted by plate. Well-level data were exported to assess the number of analyzed cells/well **(A)** and percent confluence **(B)**, whereas cell-level data were exported for the individual positional features **(C,D)**. Graphs are faceted by plate (TC0000####) and each facet represents one biological replicate (i.e., independent culture) which includes 16 technical replicates/concentration.

Supplementary Figure S3 ± Antibiotics faceted by plate. Well-level data were exported to assess the number of analyzed cells/well **(A)** and percent confluence **(B)** in R statistical software. Graphs are faceted by plate (TC0000####) and each facet represents one biological replicate (i.e., independent culture) which includes 16 technical replicates/condition.

Supplementary Figure S4 CV concentration-response curves. Cell-level data were exported to generate cell viability concentration-response curves for each chemical in R statistical software. Data represent three biological replicates (i.e., independent cultures) which includes two technical replicates/chemical concentration per biological replicate. The enlarged symbols depict the BMC for normalized cell count or cytotoxicity (PI-positive cells). The selected reference chemicals (Figure 7) are not displayed.

Supplementary Figure S5 Feature-level potency-magnitude plots. Cell-level data were exported to generate feature-level potency-magnitude plots for each chemical in R statistical software. Data represent three biological replicates (i.e., independent cultures) which includes two technical replicates/chemical concentration per biological replicate. The dotted-lines on the curves depict the global (blue), cytotoxicity (red), and normalized cell count (gray) BMC, respectively. The gray band represents the concentration-range modeled (i.e., all concentrations ≤ LOEC) in the horizontal direction and ± 1 standard deviation from the median of the DMSO vehicle control in the vertical direction. Selected reference chemicals (Figure 7) are not displayed.

Supplementary Figure S6 Global concentration-response curves. Cell-level data were exported to generate global concentration-response curves for each chemical in R statistical software. Data represent three biological replicates (i.e., independent cultures) which includes two technical replicates/chemical concentration per biological replicate. The enlarged symbols depict the BMC for phenotypic profiling (see Table 2). Selected reference chemicals (Figure 7) are not displayed.
