# Supplementary figures and images for: Optimization of Human Neural Progenitor Cells for an Imaging-Based High-Throughput Phenotypic Profiling Assay for Developmental Neurotoxicity Screening
Source: Front Toxicol. 2022 Feb 16;3:803987. doi: 10.3389/ftox.2021.803987 (PMC8915842; doi:10.3389/ftox.2021.803987)

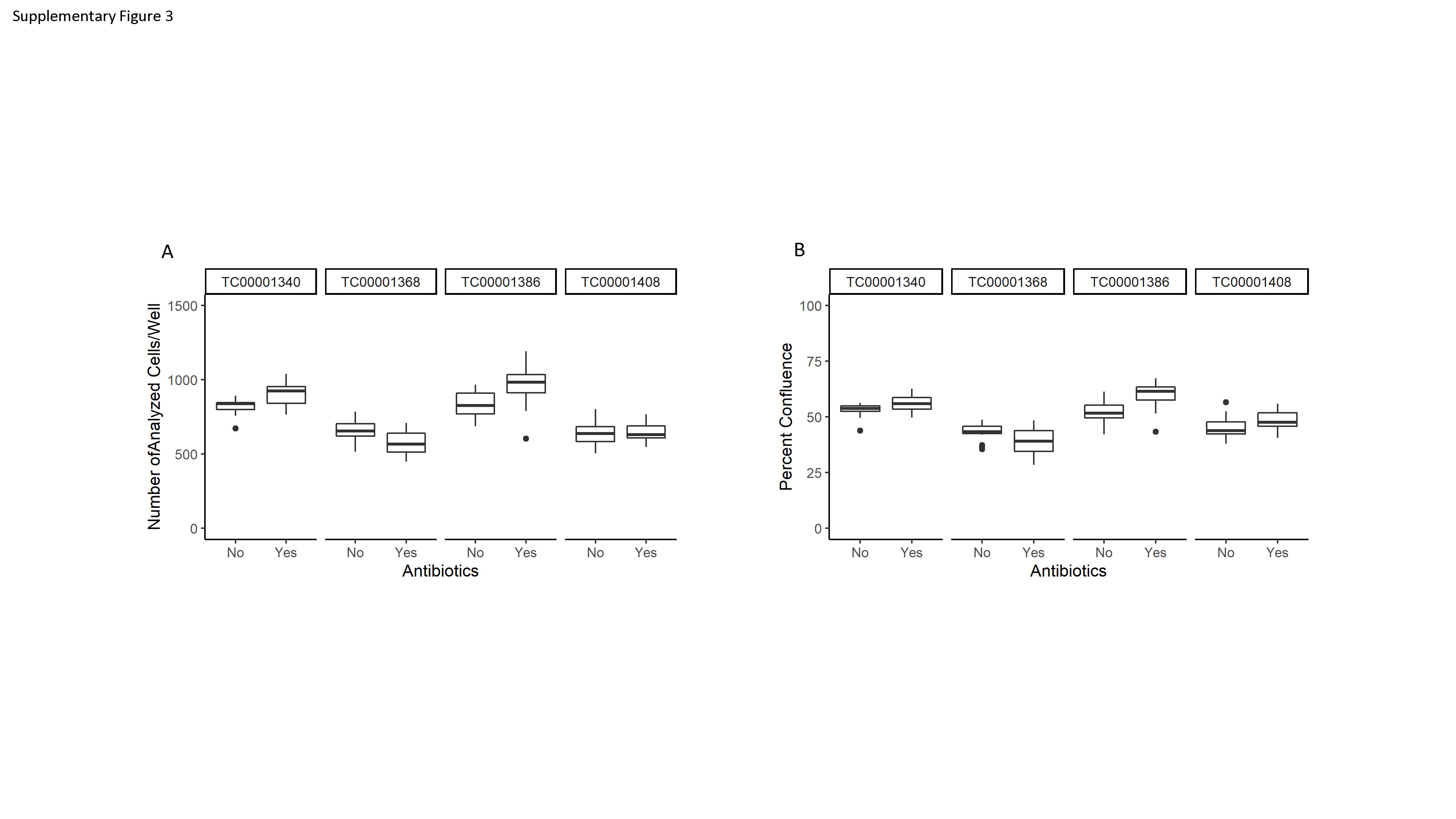

Supplement: Supplementary file 1 [file Image3.JPEG]

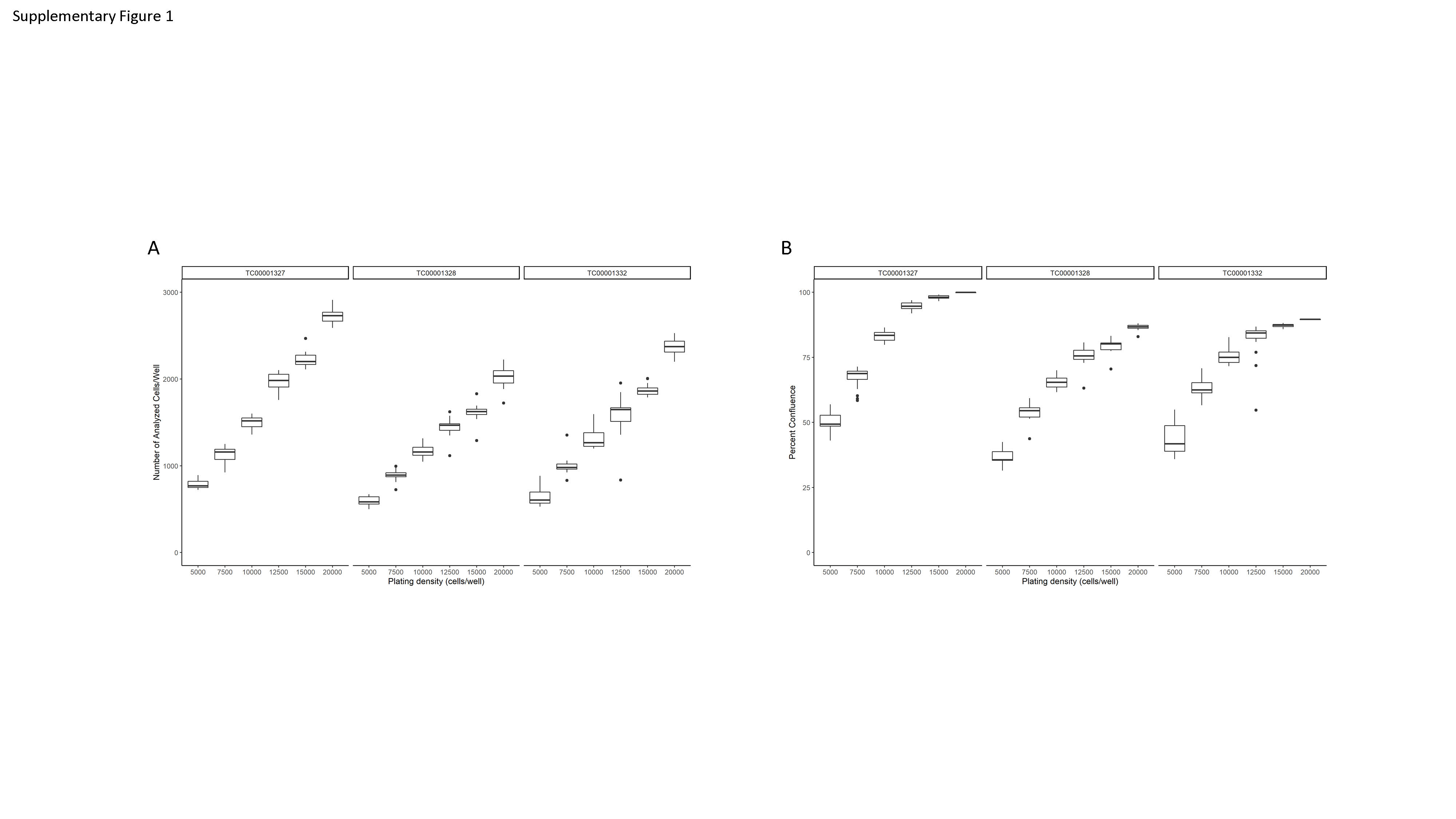

Supplement: Supplementary file 2 [file Image1.JPEG]

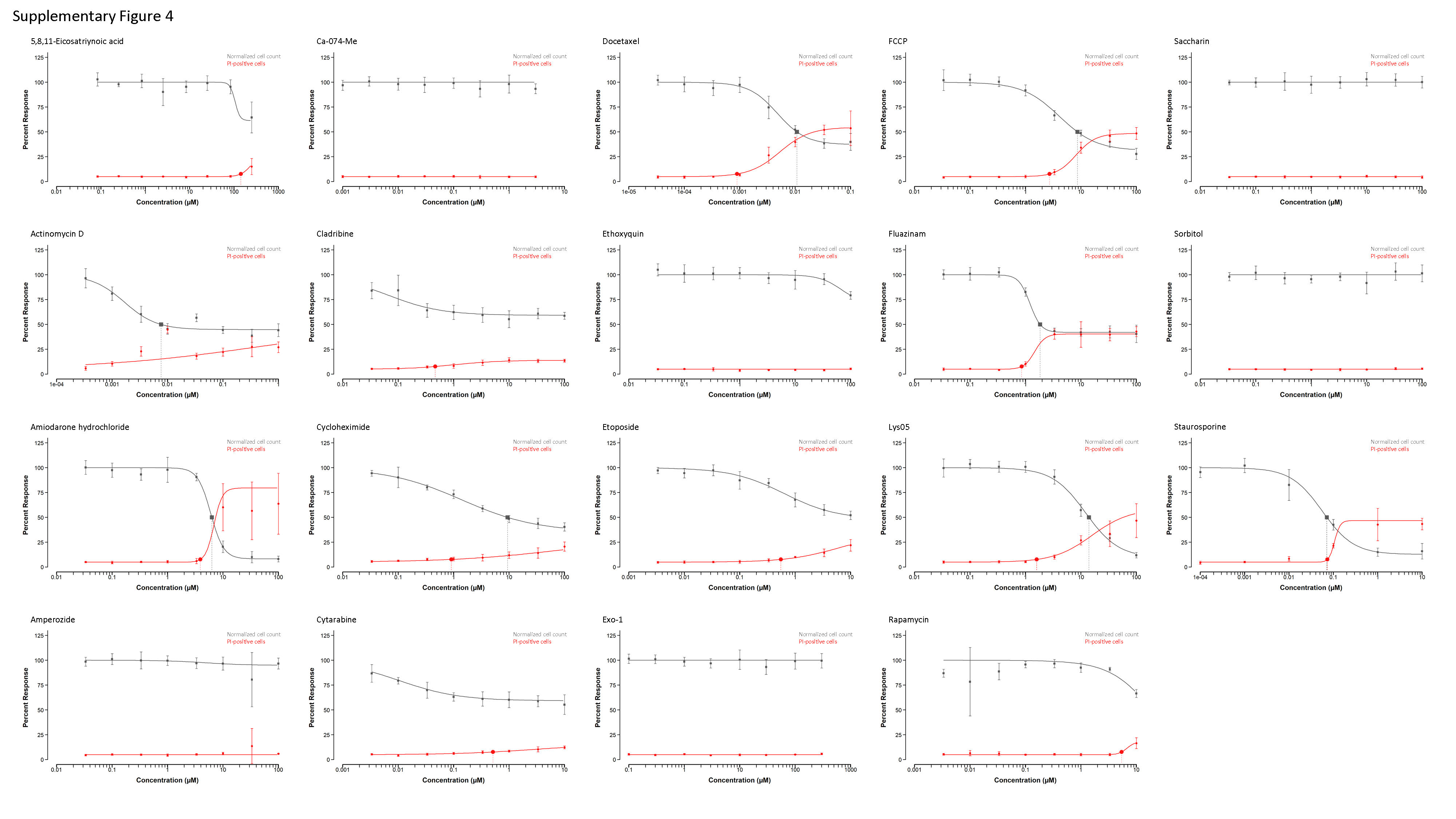

Supplement: Supplementary file 3 [file Image4.JPEG]

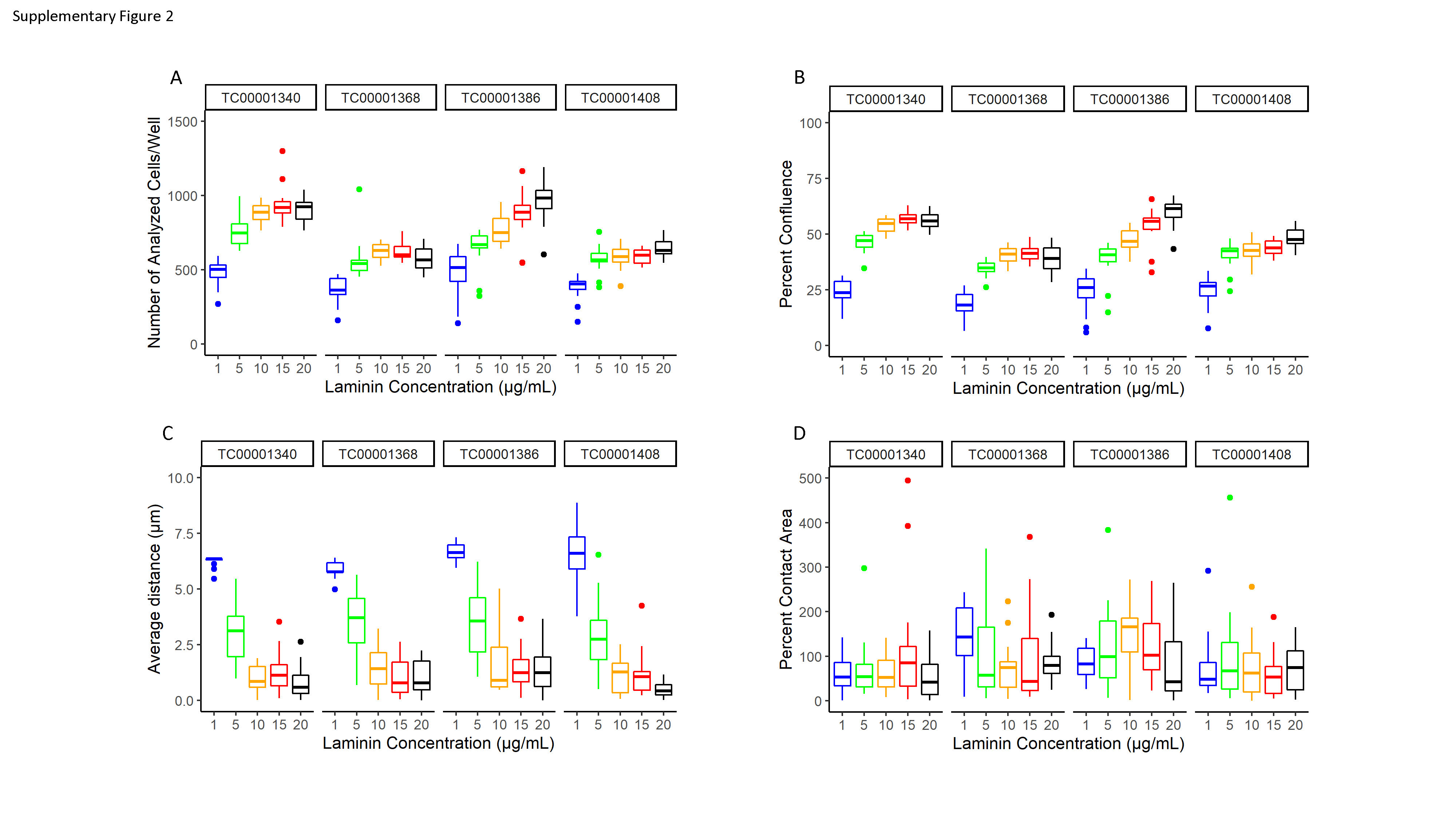

Supplement: Supplementary file 4 [file Image2.JPEG]

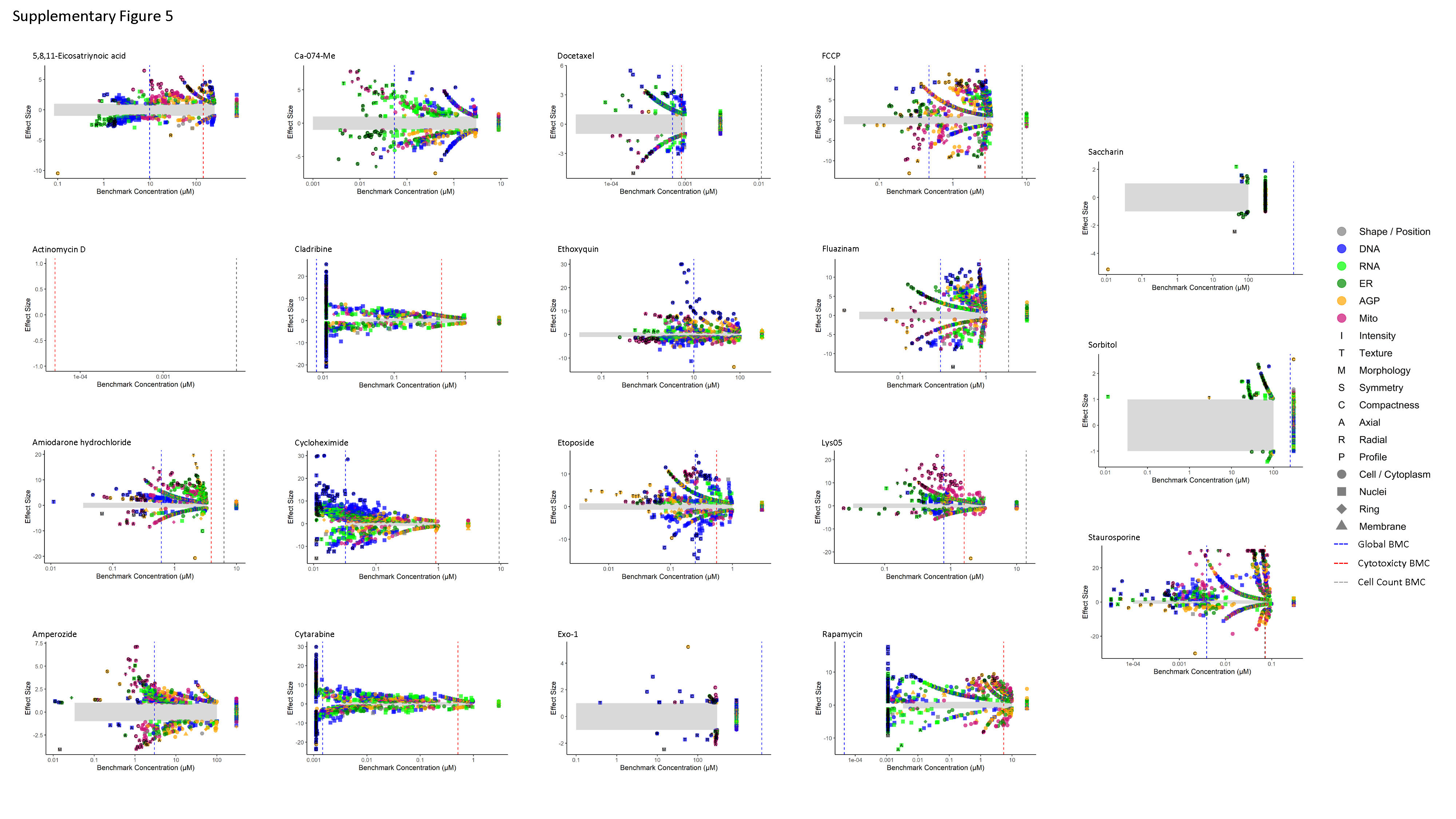

Supplement: Supplementary file 5 [file Image5.JPEG]

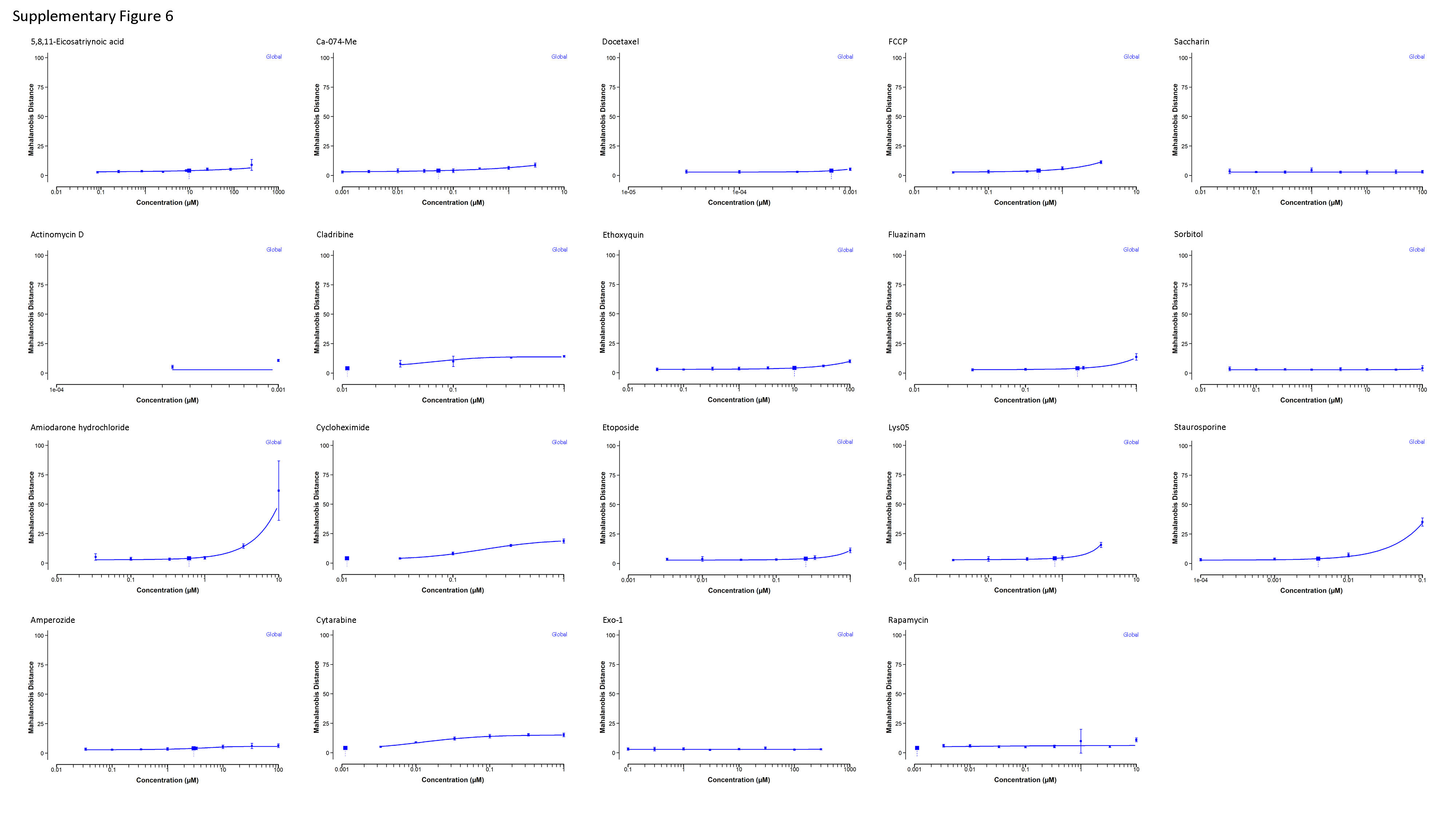

Supplement: Supplementary file 6 [file Image6.JPEG]
